# Spin noise explores local magnetic fields in a semiconductor

**DOI:** 10.1038/srep21062

**Published:** 2016-02-17

**Authors:** Ivan I. Ryzhov, Gleb G. Kozlov, Dmitrii S. Smirnov, Mikhail M. Glazov, Yurii P. Efimov, Sergei A. Eliseev, Viacheslav A. Lovtcius, Vladimir V. Petrov, Kirill V. Kavokin, Alexey V. Kavokin, Valerii S. Zapasskii

**Affiliations:** 1 St.-Petersburg State University, Spin Optics Laboratory, Peterhof, St.-Petersburg 198504, Russia; 2 Ioffe Institute of the RAS, Sector of quantum coherent phenomena, St.-Petersburg 194021, Russia; 3 St.-Petersburg State University, Resource Center “Nanophotonics”, Peterhof, St.-Petersburg 198504, Russia; 4 Ioffe Institute of the RAS, Laboratory of Semiconductor Optics, St.-Petersburg 194021, Russia; 5 University of Southampton, Department of Physics & Astronomy, Southampton SO17 1BJ, United Kingdom

## Abstract

Rapid development of spin noise spectroscopy of the last decade has led to a number of remarkable achievements in the fields of both magnetic resonance and optical spectroscopy. In this report, we demonstrate a new – magnetometric – potential of the spin noise spectroscopy and use it to study magnetic fields acting upon electron spin-system of an *n*-GaAs layer in a high-Q microcavity probed by elliptically polarized light. Along with the external magnetic field, applied to the sample, the spin noise spectrum revealed the Overhauser field created by optically oriented nuclei and an additional, previously unobserved, field arising in the presence of circularly polarized light. This “optical field” is directed along the light propagation axis, with its sign determined by sign of the light helicity. We show that this field results from the optical Stark effect in the field of the elliptically polarized light. This conclusion is supported by theoretical estimates.

Spin noise spectroscopy (SNS), primarily demonstrated on atomic systems^1^,^2^,^3^, has recently attracted significant interest mainly due to its application to semiconductor structures, where this technique proved to be most efficient^4^,^5^,^6^. The basis of the SNS is provided by the fluctuation-dissipation theorem, which implies possibility of detecting resonances of linear susceptibility of the medium without its excitation, by “listening” to a noise of the medium in its equilibrium state. As applied to magnetic resonance spectroscopy, this principle can be realized by detecting fluctuations of the Faraday rotation for the probe beam passing through a transparent (at the probe wave frequency) paramagnet in a magnetic field directed across the light beam propagation direction. These fluctuations are proportional to fluctuations of the medium magnetization. In this configuration, the Faraday rotation noise spectrum reveals a peak at the magnetic resonance frequency corresponding to precession of spontaneous fluctuations of the spin ensemble at the Larmor frequency. Since this technique, unlike conventional electron spin resonance (ESR) spectroscopy, does not imply excitation of the resonance, it is considered to be essentially nonperturbative. Along with this property, which was initially regarded as the most important merit of SNS, the new technique has revealed a number of capabilities inaccessible either to conventional ESR spectroscopy, or even to linear optical spectroscopy in general^7^,^8^,^9^,^10^. In particular, it allows one making ESR measurements in a wide range of frequencies extending far beyond the bandwidth of optical detectors (up to THz) with no special microwave equipment, to identify statistics of spin carriers, to perform three-dimensional tomographic measurements with a fairly high spatial resolution, to penetrate inside inhomogeneously broadened bands. A highly important advancement of the SNS was related to the introduction of the Fourier-transform-based technique of spin noise (SN) processing, which has dramatically improved sensitivity of the method^11^, making it possible, for instance, to measure spin noise of a single spin^12^ or to detect nuclear spin fluctuations^13^. Thanks to all these opportunities and in combination with almost nonperturbative character of the measuring procedure, the SNS has made it possible to perform many fascinating experiments (see ref. 14 for review) and thus has turned, nowadays, into a highly useful and, in many respects, unique method of research in the field of magnetic resonance and spin dynamics in semiconductors.

Application of the SNS to semiconductors and, in particular, to semiconductor nanostructures, such as quantum wells and quantum dots, often requires further improvement of the polarimetric sensitivity. It can be achieved either by direct increase of the probe beam power^15^,^16^ or by placing the sample into a Fabry-Perot cavity^17^. In these studies, it became clear that, in practice, SNS cannot remain perfectly nonperturbative. This fact, although hinders nonperturbative measurements, may be useful for studying dynamics of non-equilibrium spin systems and thus provide the basis of the nonlinear SNS. Examples of the nonlinear SNS have already been given by the experimental studies^11^,^16^,^18^,^19^,^20^ in which the effects of probe beam intensity on the detected spin noise spectra were noticed. A spectacular example of the nonlinear SNS has been reported in the recent work^21^, where the elliptically polarized probe beam in the region of nominal transparency of the sample (*n*-doped GaAs) has made it possible to optically orient the host lattice nuclear spin system of the semiconductor in the Voigt geometry. This is possible if additional fields, besides external one, break the pure Voigt geometry of the system^22^.

It is known that the electron spin noise (SN) spectra at arbitrary orientation of the external magnetic field generally reveal two components^7^,^23^. One of them is centered at zero frequency and reflects fluctuations of the longitudinal (with respect to the applied magnetic field) magnetization, while the other, at Larmor frequency, results from fluctuations of the transverse magnetization. Relative contributions of these two components are controlled by mutual orientation of the light beam and magnetic field and therefore can be used to monitor direction of the effective magnetic field acting upon the spin system under particular experimental conditions. This fact distinguishes the SN-based magnetometry from the one based on conventional magnetic-resonance and may essentially contribute to potentialities of the methods of optical magnetometry^24^.

In this report, we use the spin noise spectroscopy to study mechanisms of nonlinear interaction of elliptically polarized probe light of sub-bandgap energy with electron spin system of the *n*-GaAs crystal. The main attention is devoted to an analysis of properties of the effective field, termed hereafter as *optical* magnetic field, created by circularly polarized component of the probe beam and revealed in a highly spectacular form in the SN spectrum of the sample. We consider origination of the optical field and show that it most likely results from the probe-helicity-dependent optical Stark effect relevant at high power densities of the electromagnetic field inside the microcavity even for the medium transparent at the appropriate wavelength. A theory of such an effect for bulk microcavities is developed.

## Results

### Experimentals

**Figure 1:**
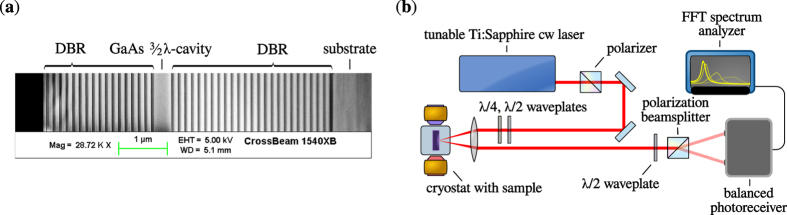
Sample and setup. SEM image of the studied structure (**a**) and schematic of experimental arrangement (**b**).

We used a 3*λ*/2-layer of *n*-type GaAs (*n* ≈ 4 × 10^16^ cm^−3^) embedded into a graded high-Q (quality factor *Q* ~ 10^4^) microcavity with GaAs/AlAs Bragg mirrors (Fig. 1a). The detected spin noise was provided by delocalized electrons of the conduction band. The standard spin noise spectroscopy setup was modified to apply elliptically polarized light and static magnetic fields both in Voigt and Faraday geometries, Fig. 1b. The details about the sample and the experimental setup can be found in refs 20,21,25 and in Methods section. In our experiments the wavelength of the probe 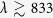 nm beam corresponded to the region of nominal transparency of the sample, *λ* > *λ*_*g*_, where *λ*_*g*_ ≈ 820 nm corresponds to the fundamental absorption edge of GaAs. Thus, the nonlinear effects in the SNS of the microcavity sample observed at relatively low intensities of the probe beam arise due to high power density of the light field inside the microcavity.

We observed two types of the light-induced nonlinear effects in this system: those with temporally retarded response related to dynamic nuclear spin polarization and nuclear spin dynamics, similar to that reported in ref. 21, and those arising instantaneously within accessible time resolution (see below) and related, in particular, to the optical magnetic field.

### Retarded response

**Figure 2:**
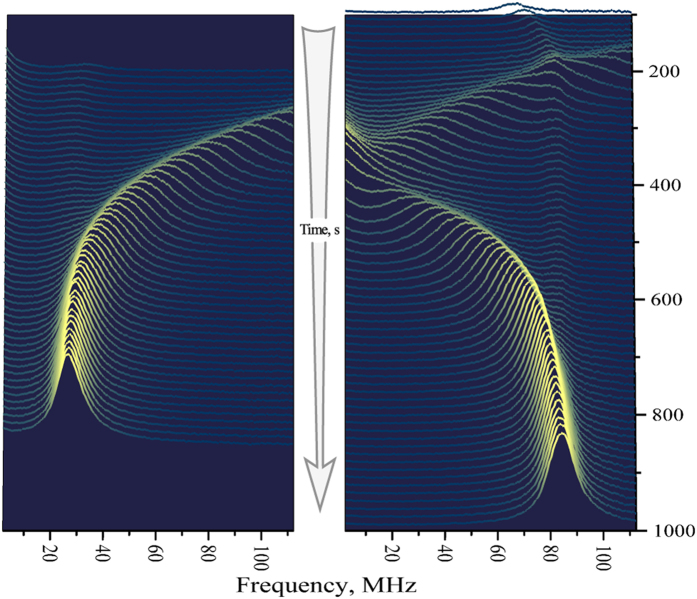
Nuclear spin relaxation in the SN spectrum. Time evolution of the SN spectrum after pumping the sample, for several minutes, by circularly polarized probe beam in a longitudinal magnetic field. The two panels correspond to different signs of circular polarization with the same sign of the applied longitudinal field. Plots are shifted in vertical direction for visual convenience. Accumulation time for each curve is ~1 s. Time interval between two successive recordings is ~13 s. *T* = 5 K.

Effects of the first type, in similarity with observations of preceding work^21^, were revealed in the most pronounced way after keeping the sample in the longitudinal magnetic field in the presence of circularly polarized probe beam. Under these conditions, nuclear system of the sample in the microcavity was efficiently oriented optically, and dynamics of the relaxing nuclear system, evidenced via the Overhauser field-induced time-dependent shift of the SN peak could be directly observed in dynamics of the SN spectrum in the Voigt geometry. Figure 2 shows example of such dynamics obtained in this way. The slow (retarded) response of the SN spectrum was observed as a drift of the precession peak in the field of relaxing nuclear spin system with characteristic times ~150 … 200 s. The right and left panels of Fig. 2 correspond to the signs of circular polarization and longitudinal magnetic field being the same or opposite. The faint peaks in the spectrum, which are practically time-independent are most probably related to a small unintentional doping of the first few layers in the Bragg mirrors. The nuclear spins in the barriers are weakly polarized and their spin dynamics is expected to be faster, hence the spin precession of these electrons is almost unaffected by the Overhauser field. These effects are, however, beyond the scope of the present report.

**Figure 3:**
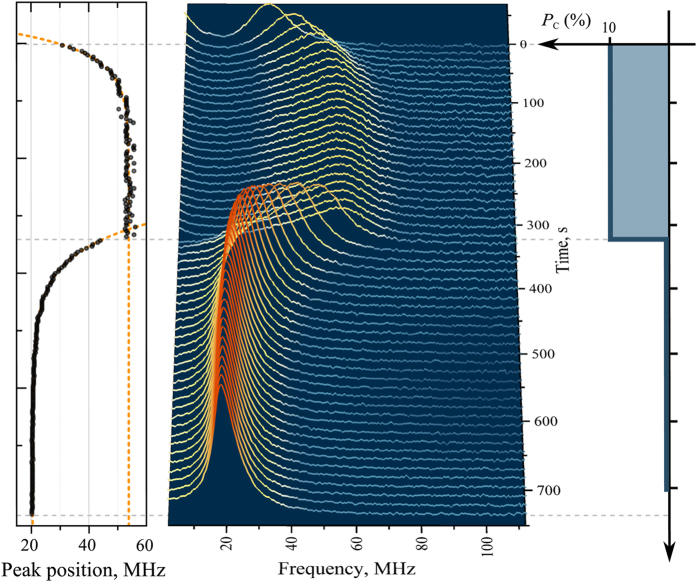
Response of the SN spectrum to switching the probe beam ellipticity on and off. Right panel shows time dependence of the degree of circular polarization of the probe beam. Left panel represents time evolution of the magnetic peak position. Orange lines are the exponential fits with the fitting parameters *τ*_1_ = 37 s and *τ*_2_ = 55 s for the rise and decay times, respectively. *T* = 5 K.

The effect of the retarded optical response, associated with nuclear spin dynamics, can be also observed in the Voigt geometry with no longitudinal magnetic field just by switching the probe beam polarization to elliptical and back to linear. In this case (see Fig. 3), we can observe dynamics of both nuclear spin polarization and nuclear spin relaxation. The fact that the relaxation times revealed in these measurements are substantially shorter than those in Fig. 2 looks quite natural if we admit possible nonexponentiality of the nuclear spin relaxation and take into account that Fig. 2 shows only the tails of the relaxation process, while Fig. 3, on the contrary, allows us to analyze only initial stage of the relaxation.

### Instantaneous response

In addition to the retarded response, already considered in previous works^21^,^26^, the time-resolved SN spectrum contained a stepwise jump, occurring simultaneously with switching ellipticity of the probe beam (*P*_*c*_) on and off. This instantaneous response was revealed as appearance or vanishing of the peak at *ω* = 0 with simultaneous shift of the precession peak to higher or lower frequencies (Fig. 3). Exactly the same effect could be achieved in experiments with additional magnetic field when *B*_*z*_ was abruptly switched on or off. Therefore, switching of the ellipticity *P*_*c*_ was equivalent to an instantaneous change of the longitudinal magnetic field acting upon the electron spins. Experimentally, the retarded and instantaneous effects could be easily separated by making rapid measurements with accumulation times much shorter than that of the nuclear spin relaxation.

Time resolution of our setup (~10 *μ*s) did not allow us to address the timescale of instantaneous response and, thus, to find out whether this effect is formed on the timescale relevant to the electron spin dynamics (~ns) or on subpicosecond scale relevant to optical transitions. Hence, to prove that the instant modifications of the SN spectrum result from the effective optical field acting on electron spin, we rely on magnetometric capacity of the SNS, namely, on the fact that the magnetic field components aligned across (*B*_*x*_) and along (*B*_*z*_) the light beam propagation contribute essentially differently to the SN spectrum.

### The spin noise-based magnetometry and “optical field”

Recall that the SN spectrum in the presence of magnetic field, containing both the transverse (*B*_*x*_) and longitudinal (*B*_*z*_) components, takes the form^23^:


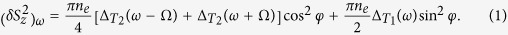


Here, *n*_*e*_ is the density of fluctuating spins, for free electrons 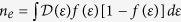, with 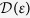 being the density of states including contributions from two spin branches and *f*(*ε*) being the distribution function^18^,^27^,^28^, *φ* is the angle between the magnetic field and *x*-axis, tan*φ* = *B*_*z*_/*B*_*x*_, the electron spin precession frequency is Ω = *gμ*_*B*_*B*/*ħ*, *g* is the electron *g*-factor, *μ*_*B*_ is the Bohr magneton, 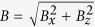, function Δ_*T*_(*x*) is defined as


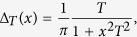


*T*_1_ is the longitudinal electron-spin relaxation time, and *T*_2_ is the transverse electron spin relaxation time. In Eq. (1), the equilibrium magnetization of electrons in the field ***B***, as well as optically induced spin polarization, are disregared.

**Figure 4:**
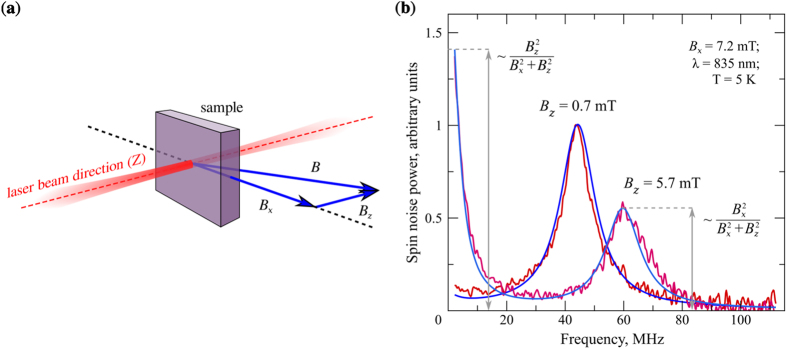
Magnetometric abilities of the SNS. (**a**) Geometry of the experiment and vector diagram illustrating magnetic fields detected by the fluctuating SN system. *B*_*x*_ is the external *transverse* magnetic field and *B*_*z*_ is the *longitudinal* magnetic field, which may be created either by an external magnet or by circularly polarized probe beam. (**b**) SN spectra obtained at fixed transverse magnetic field *B*_*x*_ for the longitudinal field *B*_*z*_ being on and off. Blue lines show the fit of the experimental data, see the text for details (0.7 mT indicated at the figure is the residual longitudinal magnetic field derived from the fitting).

Formula (1) describes the known result, already mentioned above, that, in the general case, the SN spectrum consists of two components: The one centered at magnetic resonance frequency Ω, often referred to as *magnetic* peak, and the one centered at zero frequency and called *nonmagnetic*. Spectral position of the magnetic component Ω is governed by absolute value of the total magnetic field ***B*** (Fig. 4), while the relative magnitude of the nonmagnetic component contains information about orientation of ***B*** and about its longitudinal component *B*_*z*_.

This type of behavior is illustrated by the two experimental SN spectra in Fig. 4(b) obtained, respectively, in pure Voigt geometry and in the presence of an additional static magnetic field ***B***_*z*_ created with a permanent magnet. In the presence of *B*_*z*_, one can see both appearance of the nonmagnetic (zero-frequency) component and a shift of the magnetic peak to higher frequencies. By fitting experimental data with Eq. (1), one obtains corresponding values of the external magnetic field thus confirming reliability of the method. The extracted electron-spin relaxation times are *T*_1_ ≈ 44 ns and *T*_2_ ≈ 22 ns, in reasonable agreement with literature data for similar doping level of the bulk GaAs^29^.

These magnetometric abilities of the SNS are revealed in a much more spectacular and nontrivial way in our present experiments with the elliptically polarized probe. Using the SN-based magnetometry, we observed an effective magnetic field (*optical field*) produced by circularly polarized probe beam in the *n*-GaAs microcavity.

**Figure 5:**
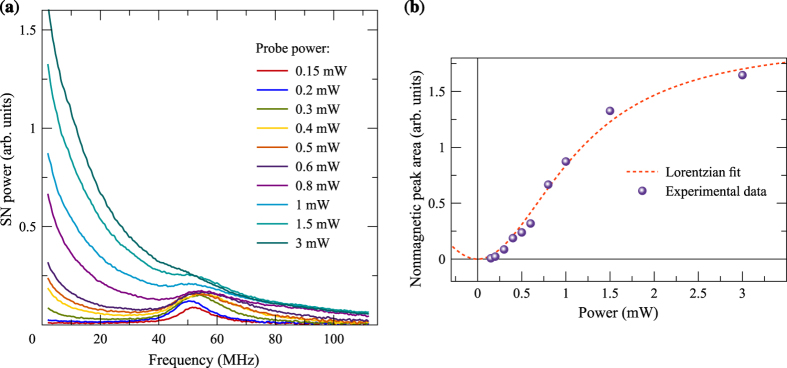
Modification of the SN spectrum with light intensity. (**a**) SN spectra recorded in the presence of the elliptically polarized probe beam (*P*_*c*_ ≈ 20%) of different intensity and (**b**) dependence of nonmagnetic component on the light beam power. The light power 1 mW in the incident beam corresponds to power density 0.3 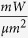 inside the cavity. Dashed line is the fit after Eq. (2). *T* = 5 K.

Experimental results presented in Fig. 5 illustrate what happens when we use an elliptically polarized probe beam instead of the beam polarized linearly. Such a configuration allows one to measure the SN spectrum in the light field with a specified helicity and, thus, to use the light beam both as a linearly polarized probe and as a circularly polarized pump. These measurements were performed fast enough, so that slow processes of nuclear spin orientation and relaxation could not noticeably contribute to results of the measurements. One can see that, with increasing intensity of the elliptically polarized probe beam, there arises the zero-frequency component of the SN spectrum. It serves as a direct evidence of presence of longitudinal component of the effective magnetic field acting on electron spins. At the same time, the magnetic component shifts to higher frequencies. Again, the experimental data can be fitted by Eq. (1) with reasonable accuracy. Example of such a comparison is shown in Fig. S1 of Supplementary Information. Using Eq. (1), we can roughly estimate this field as 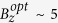 mT for the probe beam power ~0.5 mW and circular polarization degree *P*_*c*_ ≈ 20%.

Phenomenological features of the instantaneous response demonstrate fairly convincingly that it is related to light-induced modification of the magnetic field acting upon the fluctuating electron spins, rather than to light-induced changes of the electron-spin properties. The properties and nature of this *optical field **B***^*opt*^ are discussed in the next section.

## Discussion

The optical field revealed in our SNS experiments shows certain features that look nontrivial and deserve special attention.

First of all, the optical field is directed along the light propagation (*z*) axis and changes its sign with reversal of the probe beam helicity. These properties, fairly natural from the viewpoint of symmetry, were confirmed by compensating the optical field with a longitudinal field of a permanent magnet.

Second characteristic feature of the optical field is that there is no noticeable time delay between the stepwise jump in polarization of the probe and corresponding change of the SN signal. As has been shown in our additional measurements, the response of the SN signal to polarization of the probe, within our instrumental precision (~10 *μ*s), was instantaneous. In any case, possibility that the optical field ***B***^*opt*^ is of the nuclear-spin origin can be safely disregarded.

Thus, there are basically two options for the origin of the optical field. It could be caused either (i) by some electron spin system or (ii) by the electromagnetic field itself. Let us examine these options in more detail.

Note that, although the interaction between electrons is spin dependent, the electrons probed by the SNS cannot be responsible for the optical field. This follows from the Larmor theorem which states that the spin resonance frequency is not renormalized by the electron-electron interactions^30^,^31^. Deviations from the Larmor theorem can be observable only in low-dimensional semiconductor systems with spin-anisotropic interactions and, as a rule, are minor^32^,^33^. Hence, there could only be a contribution from some other electron ensemble, i.e., from electrons localized at donor pairs^34^ or from photogenerated holes.

However, the optical magnetic field is produced, in our experiments, by the light with the photon energies below the GaAs bandgap, which corresponds to the region of nominal transparency of the system. It is noteworthy also that the microcavity *Q*-factor does not show any noticeable changes with the light power (Fig. S2 of Supplementary Information). Although some absorption of light should take place to enable optical orientation of electron spins and dynamic nuclear polarization^21^,^26^,^35^,^36^, two additional spin systems mentioned above (localized electrons and photoholes) have very short spin lifetimes, typically 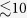 ps^37^, as compared with 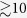 ns spin lifetimes for free electrons in our sample, and, thus, spin polarization of these spin systems should be negligible. Even if there is some electronic spin sub-system, in our sample, with sufficiently long spin lifetime (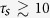 ns) to provide sizable polarization, the external transverse field *B*_*x*_ should suppress its spin *z*-component due to Hanle effect and, at the same time, give rise to the other transverse component *B*_*y*_.

The experiment has shown, however, that this is not the case: optical field *B*_*z*_ obtained from the fitting of data is independent on *B*_*x*_. Figure 5(b) presents experimental dependence of the nonmagnetic peak area on intensity of the *elliptically* polarized probe beam (*P*_*c*_ ≈ 20%) at a fixed value of the external transverse magnetic field *B*_*x*_. This dependence completely coincides with our theoretical predictions for the optical field (see below) and, thus, makes it possible to quantitatively evaluate, with sufficiently high accuracy, magnitude of this field.

Indeed, it follows from Eq. (1) that the ratio of the area of nonmagnetic component *A*_0_ to the total area *A* is simply given by (we restrict integration by positive frequencies only, in which case 
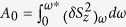
 with 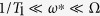)


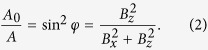


Taking into account that the optical field is parallel to *z* axis, and that its magnitude is proportional to both incident light helicity *P*_*c*_, and intensity, *I*,


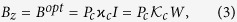


where 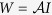 is the total light power in the illuminated spot, with 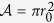 being the area of the spot with *r*_0_ ≈ 15 *μ*m being the spot radius, 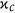 and 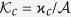 are constants. Making use of the fact that *B*_*x*_ is just the external field and fitting the data in Fig. 5(b) by Eq. (2), we obtain 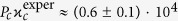 mT·*μ*m^2^/mW or


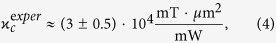


or 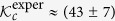 mT/mW.

Note that relative sign of the optical field *z*-component, *B*_*z*_, and circular polarization, *P*_*c*_, can be established from (i) sign of the nuclear spin temperature (determined from the temporal dependence of the electron spin precession peak position in Fig. 2, see ref. 21) and (ii) mutual orientation of the external static and optical fields: ***B*** and ***B***^*opt*^ are parallel in Fig. 2(a) and anti-parallel in Fig. 2(b).

To identify the microscopic origin of the optical field, we note that the irradiation of the quantum systems in the transparency region results in the renormalization of the electron energy spectrum^38^. This class of effects is known as *ac* or *dynamic* Stark effects, see ref. 39 for brief review. Particularly, circularly polarized radiation results in the effective Zeeman splitting of electron spin states^40^, sometimes referred to as *ac* Zeeman effect^41^,^42^. The effect of circularly polarized light can be thus considered as the generation of the effective *optical* magnetic field, just like the propagation of circularly polarized light in transparent media results in its magnetization, termed as inverse Faraday effect^43^,^44^. By symmetry reasons in cubic crystals like GaAs (*T*_*d*_ point symmetry group) this effective magnetic field can be presented in the linear in the light intensity *I* = *c*|*E*|^2^/(2*πn*_*b*_) regime as


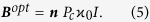


Here *n*_*b*_ is the background refractive index and *E* is the amplitude of the field, ***n*** is the unit vector in the direction of light propagation and 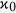 is a coefficient, which, in general, depends on the light intensity. Symmetry properties of the optical field ***B***^*opt*^ introduced in this way directly correspond to the experimental observations reported above. To evaluate the optical field we use the second-order perturbation theory, and take into account the enhancement of the radiation intensity in the microcavity due to multiple reflections from the Bragg mirrors, which yields the accumulation of the electromagnetic field in the cavity. The calculation presented in the Supplementary Information results in the field enhancement factor *f* ≈ 15 for the structure under the study. As a result, 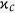 in Eq. (3) reads


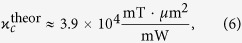


or 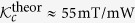. We note good agreement both in magnitude and in the formation sign between this simple estimation and the experimental value, Eq. (4).

The mechanism of the optical field formation discussed here is related to renormalization of the electron energy spectrum of bulk GaAs in the field of elliptically polarized light wave and to enhancement of the electromagnetic field intensity in microcavities. Besides, semiconductor microcavities demonstrate a variety of specific polarization-dependent linear and nonlinear effects^45^,^46^,^47^,^48^, including polarization conversion, self-induced Larmor precession, second harmonic generation, spin to angular momentum conversion, etc. Each of these effects may be of particular importance for the optical field induction in the strong coupling regime. The finding of a microscopic mechanism responsible for the optical field formation and nuclear spin pumping in microcavities constitute a fascinating new research problem that will be in scope of our further studies.

## Conclusion

In this report we used spin noise based magnetometry to reveal the “optical” magnetic field, an effective magnetic field acting on electron spins caused by the circularly or elliptically polarized light. This field arises when the light propagates through the sample in the region of transparency, scales linearly with radiation intensity and reverses its direction when the light helicity is reversed. The experiments were carried out on the bulk *n*-GaAs layer embedded into a high-*Q* microcavity, which strongly enhances the intensity of incident radiation and makes optical field pronounced. The magnetometric abilities of the spin noise spectroscopy technique allowed us to characterize this optical field quantitatively. Calculations of the field magnitude, based on the proposed model of the optical Stark effect in the field of circularly polarized light, well correlate with the experimental data.

The demonstration of the strong optical field in bulk semiconductor opens ways for coherent manipulation of the spin states like realized recently for quantum dots^49^,^50^ and for realization of non-trivial spin structures similar to those discussed for two-dimensional systems^51^,^52^,^53^.

Besides demonstrating informative potentialities of the spin noise spectroscopy, these results, in our opinion, are important for understanding the effect of optical nuclear orientation observed in microcavities. However, the mechanism of the efficient transfer of angular momentum from the light to nuclear spin system under highly unfavorable conditions when the pumping light beam acts upon the system in the region of its nominal transparency remains unsolved and intriguing problem to be analyzed in more detail elsewhere.

## Methods

### Sample

Bragg mirrors of the sample were made of GaAs/AlAs, rather than AlGaAs/GaAs^21^, pairs of layers. This allowed us to obtain approximately the same *Q*-value of the cavity as in ref. 21 with smaller number of layers: 25 and 17 pairs for the first and second mirrors (starting from the side of substrate). The room-temperature doping ~4 × 10^16^ cm^−3^ is slightly above the point of insulator-to-metal transition of the GaAs and provides the longest spin relaxation times^29^,^54^,^55^. The detected spin noise is provided by electrons with energies in the vicinity of the Fermi level since (i) the magnitude of the SN grows linearly with temperature (the behavior typical for free electrons in GaAs^18^) and (ii) the precession peak in the SN spectrum corresponds to the electron Larmor frequency in GaAs with the *g*-factor |*g*| ≈ 0.44 (see below for details).

### Experimental setup

Magnetic fields up to 0.7 T and low temperatures down to 3 K were created by the Montana Cryostation system with magneto-optic module. The output emission of a continuous wave Ti:Sapphire laser “T&D-Scan” tuned to the cavity photon mode (at the wavelength region between 833 and 840 nm) was used as a probe. The beam was focused to a spot of about 20 *μ*m in diameter on the sample surface. Polarization noise of the beam, reflected from the sample in nearly autocollimation geometry, was detected by a balanced photoreceiver with the bandwidth ~220 MHz and processed with a broadband FFT spectrum analyzer. As a result, the measured signal 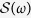 was proportional to the frequency spectrum of Kerr rotation angle fluctuation 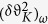. Since *δϑ*_*K*_ and electron spin fluctuation *δS*_*z*_ are directly proportional, we obtain


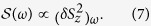


Hereafter, we use the coordinate frame with the *z*- and *x*-axes being, respectively, the light propagation direction and the axis of the transverse magnetic field.

As compared with standard SNS configuration, the experimental set-up was modified to simultaneously use the probe beam as the circularly polarized pump. A quarter-wave plate introduced into the linearly polarized beam for this purpose allowed us to control the light ellipticity. Besides, in some cases, we applied to the sample an additional magnetic field of several tens of mT directed along the light beam propagation axis to increase the pumping efficiency. For this purpose, we usually employed a permanent magnet.

## Additional Information

**How to cite this article**: Ryzhov, I. I. *et al.* Spin noise explores local magnetic fields in a semiconductor. *Sci. Rep.* **6**, 21062; doi: 10.1038/srep21062 (2016).

## Supplementary Material

Supplementary Information
