# Supplementary material for: Spin noise explores local magnetic fields in a semiconductor
Source: Sci Rep. 2016 Feb 17;6:21062. doi: 10.1038/srep21062 (PMC4756372; doi:10.1038/srep21062)
Supplement: Supplementary Information [file srep21062-s1.pdf]

# Supplementary information for:
# Spin noise explores local magnetic fields in a semiconductor

I. I. Ryzhov,[1] G. G. Kozlov,[1] D. S. Smirnov,[2] M. M. Glazov,[2,1] Yu. P. Efimov,[3] S. A. Eliseev,[3] V. A. Lovtcius,[3] V. V. Petrov,[3] K. V. Kavokin,[1,4] A. V. Kavokin,[1,5] and V. S. Zapasskii[1]

[1] *St.-Petersburg State University, Spin Optics Laboratory, Peterhof, St.-Petersburg 198504, Russia*
[2] *Ioffe Institute of the RAS, Sector of quantum coherent phenomena, St.-Petersburg 194021, Russia*
[3] *St.-Petersburg State University, Resource Center "Nanophotonics", Peterhof, St.-Petersburg 198504, Russia*
[4] *Ioffe Institute of the RAS, Laboratory of Semiconductor Optics, St.-Petersburg 194021, Russia*
[5] *University of Southampton, Department of Physics & Astronomy, Southampton SO17 1BJ, United Kingdom*

## I. ADDITIONAL EXPERIMENTAL DATA

Figure S1 shows the measured (green) and calculated (red) SN spectra in the absence and presence of the elliptically polarized light. Discrepacy between the experimental and calculated data may result from nonuniformity of the light spot.

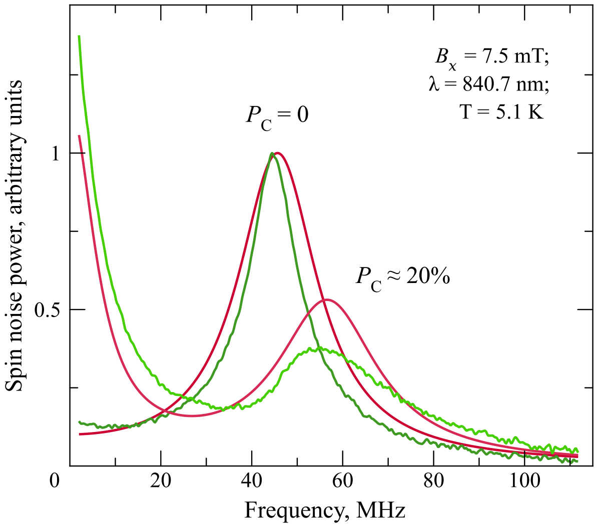


FIG. S1. Comparison of experimental shape of the SN spectrum detected in the linearly and elliptically polarized light (green curves) with the results of fitting (red curves).

Figure S2 illustrates absence of any noticeable effect of the probe beam intensity on the cavity resonance.

## II. THEORY

To evaluate the optical field we use the perturbation theory and present an effective Hamiltonian of the electron in crystal under illumination by light with the frequency $\omega$ in the second order in the light field amplitude $\boldsymbol{E}_0$ as [1, 2]

$$\delta\mathcal{H}_{n'n} = -\sum_{\alpha\beta} \chi^{\alpha\beta}_{n'n} E^*_{0\alpha} E_{0\beta}, \quad (1)$$

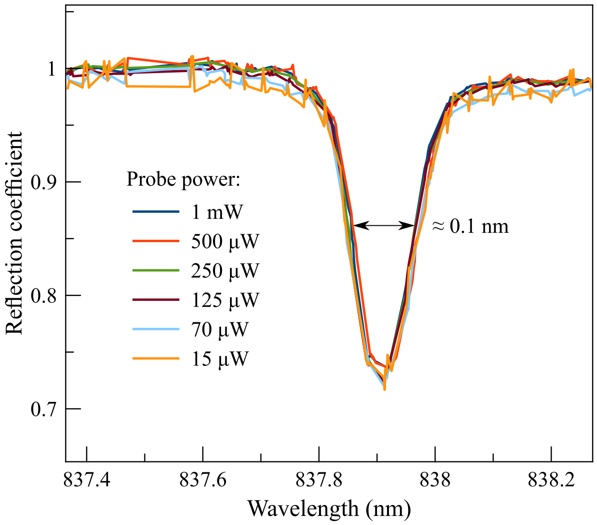


FIG. S2. Experimental plots illustrating independence of the cavity resonance width and, accordingly, the $Q$-factor on the probe beam power.

where $\alpha, \beta$ are the Cartesian components,

$$\chi^{\alpha\beta}_{n'n} = -\frac{e^2}{m_0\omega^2} \times \left[\delta_{nn'}\delta_{\alpha\beta} + \frac{1}{m_0\hbar}\sum_{m\neq n,n'}\left(\frac{p^\beta_{n'm}p^\alpha_{mn}}{\omega_{mn}-\omega} + \frac{p^\alpha_{n'm}p^\beta_{mn}}{\omega_{mn}+\omega}\right)\right], \quad (2)$$

$n, n', m, \dots$ enumerate the states (including band index, wavevector and spin), $\omega_{mn} = (E_m - E_n)/\hbar$ are the transition frequencies, $e$ is the electron charge, $m_0$ is the free electron mass, and $p^\alpha_{mn}$ are corresponding components of electron momentum operator taken between the states $m$ and $n$. For the conduction band states with the wavevector $\boldsymbol{k}$ the effective spin Hamiltonian ($s, s' = \pm 1/2$) can be written as

$$\delta\mathcal{H}_{s's} = -\chi^{\alpha\beta}_{\boldsymbol{k},s';\boldsymbol{k},s} E^*_{0,\alpha}E_{0\beta} = -\chi^{(s),\alpha\beta}_{\boldsymbol{k},s';\boldsymbol{k},s}\frac{E^*_\alpha E_\beta + E_\alpha E^*_\beta}{2} - \chi^{(a),\alpha\beta}_{\boldsymbol{k},s';\boldsymbol{k},s}\frac{E^*_\alpha E_\beta - E_\alpha E^*_\beta}{2}, \quad (3)$$

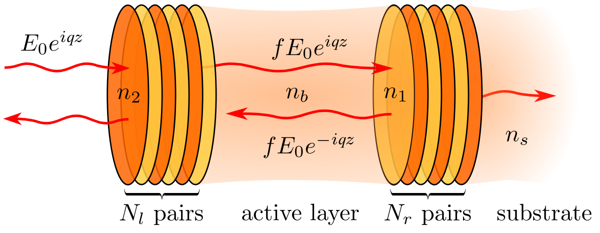


FIG. S3. Sketch of the cavity and notations.

where, to shorten the notations, we have omitted the summation over repeated subscripts and introduced symmetric and antisymmetric with respect to the permutations of $\alpha$ and $\beta$ components of the tensor $\chi$:

$$\chi^{(s),\alpha\beta}_{\boldsymbol{k},s';\boldsymbol{k},s} = \frac{\chi^{\alpha\beta}_{\boldsymbol{k},s';\boldsymbol{k},s} + \chi^{\beta\alpha}_{\boldsymbol{k},s';\boldsymbol{k},s}}{2},$$

$$\chi^{(a),\alpha\beta}_{\boldsymbol{k},s';\boldsymbol{k},s} = \frac{\chi^{\alpha\beta}_{\boldsymbol{k},s';\boldsymbol{k},s} - \chi^{\beta\alpha}_{\boldsymbol{k},s';\boldsymbol{k},s}}{2}.$$

Last term in Eq. (3) with $\chi^{(a),\alpha\beta}_{\boldsymbol{k},s';\boldsymbol{k},s}$ is sensitive to the helicity of light and in what follows we focus on this term only.

For the light propagating along $z$ axis the effective Hamiltonian, Eq. (3), can be recast in the form of Zeeman splitting

$$\delta\hat{\mathcal{H}} = \frac{1}{2} g\mu_B B_z^{opt} \hat{\sigma}_z, \qquad (4)$$

where $\hat{\sigma}_z$ is the $z$ Pauli matrix, with the optical field in the form of Eq. (5) of the main text with

$$\varkappa_0 = -\frac{4\pi\hbar^2 e^2 p_{\rm cv}^2 n_b}{3cg\mu_B m_0^2 E_g^2 \delta}. \qquad (5)$$

Here we used resonant approximation and, correspondingly, the first and the last terms omitted in Eq. (2), $n_b$ is the background refractive index, $p_{\rm cv}$ is the interband momentum matrix element, and the detuning $\delta = E_g - \hbar\omega$. Taking $E_g = 1.5$ eV, $n_b = 3.66$, $p_{\rm cv} = 1.5 \times 10^{-19}$ g·cm/s [3], $g = -0.44$ one has

$$\varkappa_0 \approx \frac{8.5 \times 10^{-5}}{(\delta/\text{meV})} \frac{\text{mT}}{\text{mW/cm}^2}.$$

In order to compare this result with experiment, one has to take into account the enhancement of light intensity inside the cavity [4, 5]. To that end, we assume that, like in experiment, the incident radiation frequency is in resonance with the cavity mode and represent the field inside the cavity in the form of two waves propagating to the left and to the right, Fig. S3:

$$\boldsymbol{E} = f\boldsymbol{E}_0 \left(e^{\mathrm{i}qz} + e^{-\mathrm{i}qz}\right), \qquad (6)$$

where $\boldsymbol{E}_0$ is the amplitude of the field outside the cavity, $\boldsymbol{q}$ is the light wavevector inside the active GaAs layer, the light is assumed to be incident on the structure from the left side, and $z = 0$ refers to the left border of the active medium ($n$-GaAs layer). The enhancement factor is

$$f = \sqrt{\frac{1 - R_l}{n_b}} \left(1 - \frac{R_l + R_r}{2}\right)^{-1} \qquad (7)$$

where $R_{l,r}$ ($1 - R_{l,r} \ll 1$) are the intensity-related reflection coefficients for the light incident from the active media on the left and right mirrors, respectively. It follows from Ref. [6] that

$$R_l = 1 - \frac{4}{n_b}\left(\frac{n_1}{n_2}\right)^{2N_l}, \quad R_r = 1 - 4\frac{n_s}{n_b}\left(\frac{n_1}{n_2}\right)^{2N_r}, \qquad (8)$$

where $n_s$ is the refraction index of the substrate, $n_1$ and $n_2$ are the refractive indices of the layers in Bragg mirrors ($n_1$ corresponds is the layers adjacent to the cavity), $N_l$ and $N_r$ are the numbers of layer pairs for the left and right mirrors, respectively, see Fig. S3 for details. We present also the quality factor of the $3\lambda/2$-cavity $Q$ defined as $Q = \bar{\omega}/(2\bar{\gamma})$, where $\bar{\omega}$ and $\bar{\gamma}$ are the real and imaginary parts of the cavity resonance frequency:

$$Q = \frac{\pi}{n_b}\left(\frac{n_1 n_2}{n_2 - n_1} + 3n_b\right)\left(1 - \frac{R_l + R_r}{2}\right)^{-1}. \qquad (9)$$

Since the number of Bragg mirrors from the side of the substrate is considerably larger than from the side of the surface, $N_r > N_l$, Eqs. (8) yield $1 - R_r \ll 1 - R_l$. So for the estimations one can simply put $R_r = 1$. In this way we obtain the relation

$$f^2 = \frac{2Q}{\pi}\left(\frac{n_1 n_2}{n_2 - n_1} + 3n_b\right)^{-1}. \qquad (10)$$

Due to the roughnesses of the interfaces, the $Q$-factor of the microcavity may be smaller than the value obtained from Eq. (9). Thus it is preferable to estimate the factor $f$ from Eq. (10) using the experimental value of $Q$, than directly using Eq. (7).

For the parameters of the structure under study: $n_1 = 2.98$, $n_2 = n_s = 3.66$, $N_l = 17$, $N_r = 25$, $Q = 8700$ the Eq. (10) gives $f^2 = 205$. Note that substantial difference between $Q$ and $f^2$ results from the effective field penetration into Bragg mirrors. Hence, $\mathcal{K}_c$ in Eq. (3) of the main text reads

$$\mathcal{K}_c^{\rm theor} = \frac{2f^2\varkappa_0}{n_b\mathcal{A}} \approx 55\,\frac{\text{mT}}{\text{mW}}, \qquad (11)$$

for the detuning $\delta = 25$ meV and probe area $\mathcal{A} = \pi r_0^2$, $r_0 = 15$ $\mu$m. We note good agreement both in the magnitude and in the sign between this simple estimation and the experimental value, Eq. (4) of the main text.

[1] P. S. Pershan, J. P. van der Ziel, and L. D. Malmstrom, Phys. Rev. **143**, 574 (1966).
[2] G. L. Bir and G. E. Pikus, *Symmetry and Strain-induced Effects in Semiconductors* (Wiley/Halsted Press, 1974).
[3] I. Vurgaftman and J. R. Meyer, Journal of Applied Physics **94**, 3675 (2003).
[4] M. S. Ünlü and S. Strite, Journal of Applied Physics **78**, 607 (1995).
[5] J. Ye and T. W. Lynn (Academic Press, 2003) pp. 1–83.
[6] E. L. Ivchenko, *Optical spectroscopy of semiconductor nanostructures* (Alpha Science, Harrow UK, 2005).
